# Prediction Model of VCA Formed by the Packing of Hybrid Lithological Coarse Aggregates Used in SMA

**DOI:** 10.3390/ma15248952

**Published:** 2022-12-14

**Authors:** Weiliang Jiang, Long Liu, Weidong Cao, Shanglei Yang, Shutang Liu, Jingchen Li

**Affiliations:** 1 Shandong Hi-Speed Infrastructure Construction Co., Ltd., Jinan 250001, China; 2 School of Qilu Transportation, Shandong University, Jinan 250002, China; 3 Shandong Hi-Speed Zilin Expressway Co., Ltd., Zibo 255100, China

**Keywords:** voids in coarse aggregate (VCA), basalt and lime coarse aggregates, prediction model, aggregate gradation, stone matrix asphalt (SMA)

## Abstract

The voids in coarse aggregate (VCA) is an important volumetric index in the mineral aggregate gradation design of stone matrix asphalt (SMA) mixtures. To explore the law of variation for VCA formed by the packing of basalt and lime coarse aggregates, a uniform design method and vibrating compaction tests were used to establish the prediction model. Based on the test results and stepwise regression analysis, a reliable prediction model of VCA was obtained. There is a multiple nonlinear relationship between the VCA and the proportion of each coarse aggregate in the mixture. Regardless of the type of coarse aggregates used, the rule of VCA with different forms of aggregate gradation curves has universal significance. This conclusion can help to determine the aggregate gradation in the design of SMA mixtures.

## 1. Introduction

Stone matrix asphalt (SMA) is a typical skeleton-dense mixture comprising more than 70% coarse aggregates [1]. The physical and mechanical properties of coarse aggregates determine the pavement and service performance of SMA, especially the high-temperature resistance and anti-skidding properties. The use of basalt and diabase continues to rise in China because of their high mechanical strength. Due to the increased demand for SMA and limited aggregate supply, low-cost aggregates have gained more attention from environmental and economic views. Given this background, some scholars have carried out research on SMA preparation by replacing (in whole or in part) basalt aggregates with low-cost aggregates, such as limestone. Li et al. [2] examined the high-temperature stability of SMA with limestone and basalt aggregates. According to the rutting and Rotary Loaded Wheel Tester or Rutmeter (RLWT), SMA-13 comprised of high-quality aggregates had higher dynamic stability, better performance under traffic loads, and more adhesion between limestone and asphalt. By contrast, limestone SMA has poor strength, and partial breakage often occurs inside mixtures, so it is hard to sustain heavy traffic loads. Hussain et al. [3] evaluated the volumetric parameters and performance of SMA mixtures with different proportions of limestone and basalt coarse aggregates. Test results showed that the SMA had excellent strength and durability when the two lithological coarse aggregates were mixed in a 1:1 ratio. Li [4] prepared SMA with unmodified asphalt and limestone, and compared its performance to the basalt SMA. The results indicated that limestone SMA was a less effective interlock than basalt SMA, while it had better economic benefits. Iskender [5] investigated the rutting resistance of basalt and basalt–limestone aggregate combinations for coarser and finer SMA mixtures. Four lithological combinations were designed using basalt as a coarse aggregate and basalt and limestone aggregates as fillers and fine aggregates. It was concluded that the rutting resistance of the SMA mixture relatively decreased with the incorporation of limestone aggregate in mineral gradation as a fine or filler aggregate. However, the study had great importance for the shortage of basalt aggregate quarries. Due to the shortage and high price of basalt, the feasibility of utilizing limestone in porous asphalt concrete (PAC) was studied by Hu et al. [6]. The results showed that PAC with limestone was a prospective lower layer of the double-layer porous asphalt pavement. Ibrahim et al. [7] replaced basalt aggregates with different proportions of limestone aggregates in asphalt concrete mixes. The results indicated that the comprehensive performance of pavement was optimal when basalt and limestone were, respectively, selected as coarse and fine aggregates. Additionally, 20% of the filler portion of the aggregate was replaced by lime to solve the potential stripping of the optimal mix. The effect of aggregate properties on the stripping and creep behavior of hot-mix asphalt (HMA) was investigated by Abo-Qudais et al. [8]. They found that unconditioned HMA with basalt aggregate resists creep better than those with limestone; however, the conditioned mixes with basalt were less resistant to creep strain than those with limestone aggregate. Cao et al. [9] compared the performance of three kinds of SMA mixtures named B-SMA, L-SMA, and BL-SMA, which were prepared using basalt coarse and fine aggregates, limestone coarse and fine aggregates, basalt coarse aggregates, and limestone fine aggregates, respectively. They concluded that B-SMA showed the best rutting resistance, followed by BL-SMA, and finally L-SMA, while the reverse order was shown in cracking resistance and moisture susceptibility. Yi [10] and Huang et al. [11] also concluded that limestone SMA had better low-temperature performance and water stability, though its dynamic stability was weaker than that of basalt SMA. Above all, numerous studies have been conducted on SMA mixtures with different lithological aggregates. It has been demonstrated that the partial application of limestone and other low-cost stones in SMA was feasible, and some valuable conclusions were obtained. However, further studies are needed to design and optimize SMA gradation for various lithological aggregates. For example, the same gradation is often applied when comparing the performance of SMA mixtures using basalt and limestone aggregates. It is inappropriate to simply attribute the resulting differences in volume indexes (e.g., VMA) and performance (e.g., rutting resistance) of two SMA mixtures to different lithological aggregates. Coarse aggregates with different lithology have different angularity and densities. The same gradation curve will inevitably produce different volume indexes such as VMA, and it is possible that the mixture’s performance, such as high-temperature performance, will be different, or inevitable. Therefore, SMAs of various lithological aggregates should have respective appropriate gradation. It is necessary to conduct gradation design for various lithological aggregates separately.

As is well known, one of the most distinctive features of an SMA is the gap-graded aggregate structure with a stone-on-stone skeleton. The voids in coarse aggregate (VCA) and the voids in coarse aggregate of asphalt mixture (VCA_mix_) are the critical volume indicators relating indirectly to the gradation design. The standard method to evaluate the stone-on-stone aggregate skeleton of SMA is by comparing the values of VCA and VCA_mix_ [12,13]. Miranda et al. [14] proposed an SMA design methodology based on an analytical design approach to optimize the stone-on-stone effect. In the methodology, considering the occurrence of the voids and the particle breakage in the field, the bulk density of compacted coarser aggregates was evaluated. Meanwhile, the voids in the mineral aggregate (VMA) plays a vital and screening role in the aggregate grading designs. Additionally, the VCA_mix_ provides a maternal space for VMA and partly restricts the VMA value [15,16]. Previous research indicated that an intrinsic relationship existed between VMA and VCA, the percentage passing of the boundary sieve, the bulk specific gravity of the mineral aggregate, etc. [17,18]. Therefore, studying the VCA of coarse aggregates is fundamental and necessary for the grading design of SMA and HMA. We have researched the statistical law of VCA formed from the packing of coarse basalt aggregates and established four effective regression models [19]. However, there are few studies on the statistical law of VCA formed by different lithological coarse aggregates. In this paper, based on a uniform experimental design method and vibrating compaction experiments of various lithological coarse aggregates used in SMA13, the prediction models of VCA formed by the hybrid coarse aggregates (mixed lime and coarse basalt aggregates) were developed. Additionally, the relation between the VCA and the form of coarse aggregate grading curve was analyzed, which helps to guide aggregate grading design for SMA mixtures with different lithological aggregates.

## 2. Materials and Methods

### 2.1. Materials

**Figure 1:**
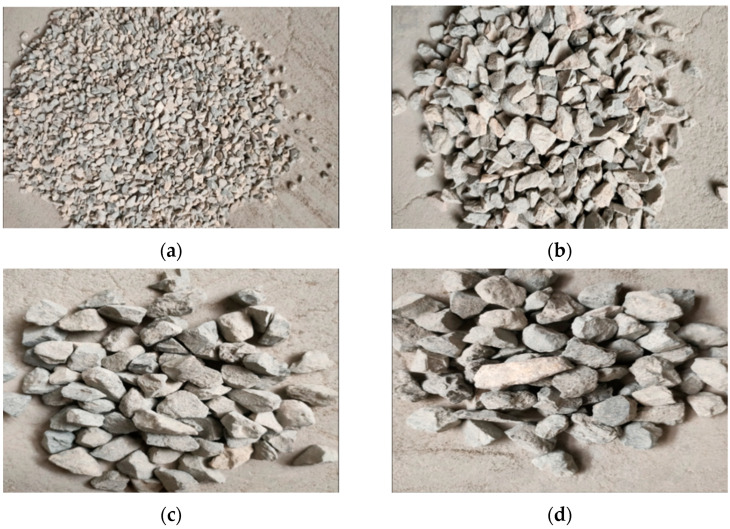
Two kinds of lithological coarse aggregates. (**a**) 2.36–4.75 mm limestone, (**b**) 4.75–9.5 mm limestone, (**c**) 9.5–13.2 mm basalt, and (**d**) 13.2–16 mm basalt.

Currently, in expressway engineering in China, the nominal maximum size 13.2 mm SMA mixture is the most commonly used in the surface layer of the pavement structure. In terms of Chinese specifications [13] and previous research [17,19], a 2.36 mm size can be used as a boundary sieve between coarse and fine aggregates. In order to reduce test errors, all coarse aggregates were sieved into four groups with single-size particles (13.2 to 16 mm, 9.5 to 13.2 mm, 4.75 to 9.5 mm, and 2.36 to 4.75 mm) as per China Standard T0302-2005 [20]. To ensure the better strength and stability of coarse aggregate skeleton in SMA, basalt was chosen for the aggregates of two maximum particle sizes (13.2 to 16 mm, 9.5 to 13.2 mm) and limestone for the aggregates of relative minimum particle sizes (4.75 to 9.5 mm, 2.36 to 4.75 mm), which were mixed as the coarse aggregate skeleton of hybrid lithological SMA. The basic properties and appearance of coarse aggregates are given in Table 1 and Figure 1, respectively. All aggregates meet the technical requirements of Chinese specifications [13].

### 2.2. Experiments Design and Test Methods

#### 2.2.1. Design of Experiment

The mathematical optimization method is usually used to design an experimental scheme when many factors and levels are involved in the experiment. To reduce the test workload and based on existing application experiences, the uniform design (UD) method was utilized for experimental scheme design of four coarse aggregate mixes in this study. It was proposed by Chinese mathematicians Fang Kaitai and Wang Yuan in 1978 and has been successfully applied in the petroleum, chemical, and automotive industries, which is widely acknowledged in the natural sciences field [21].

The UD method is similar to the orthogonal factorial design, using well-designed tables to carry out experimental designs. It only ensures that experimental points spread uniformly on the experimental domain but without taking the “symmetrical comparability” into account, which enables it to obtain the most information with the least number of test samples, ensuring the statistical properties of the experimental points with uniform distribution. Only one experiment is needed for each factor level, which is significantly less than orthogonal designs [21]. The uniform design table is represented by Un(p^m^) following the orthogonal table. In this table, U, n, p, and m represent the UD table number, the number of horizontal rows (i.e., the number of experiments), the number of levels of each factor, and the number of columns in the table (i.e., as many factors that can be arranged as possible), respectively. The details of the theory and application of the UD method can be seen in the literature [22]. The UD table ${\mathrm{UM}}_{16}^{*}\left(16^{4}\right)$ was applied in this experiment. Four factors x_1_, x_2_, x_3_, and x_4_, respectively, represent the proportion of 13.2–16 mm basalt, 9.5–13.2 mm basalt, 4.75–9.5 mm limestone, and 2.36–4.75 mm limestone in total hybrid coarse aggregates, with each factor taking 16 levels, and the experimental scheme is shown in Table 2.

#### 2.2.2. Test Method

**Figure 2:**
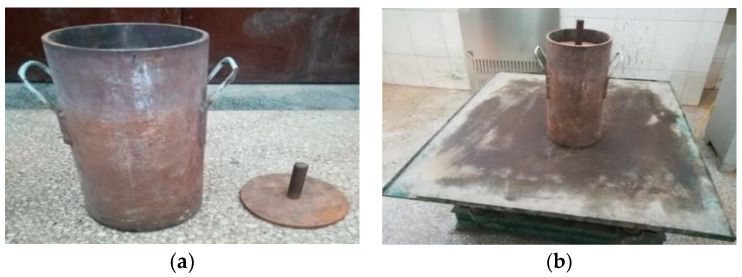
Vibrating vessel for the coarse aggregate and vibration device. (**a**) Vessel for filling the coarse aggregate. (**b**) Vibrating table.

The VCA formed by the packing of coarse aggregates can be tested by dry-rodded and vibrating compaction methods, and the results obtained with each method will be different [20]. The dry-rodded test process is greatly affected by human causes, so the automatic-controlled vibrating compaction was employed in the experiment. The test equipment includes the vibrating table with controller, a 10-litre iron vessel, and a circular weight stack (the diameter is slightly smaller than the inner diameter of the vessel) with a grip, shown in Figure 2. The test process was as follows:

1. A total of 16 kinds of hybrid coarse aggregate mixture samples were prepared in accordance with the scheme in Table 2, which must be well-mixed.
2. Each mixed sample should be poured into the vessel in three parts. The amount of mixture poured for the first part was estimated according to the height of the mixture after pouring, which was about 1/3 the vessel height minus the reserved 5 cm of the top. Then, the surface was levelled, a weight stack was set in place, and the vibrating table was initiated with a frequency of 50 Hz and 2 min of compaction time. After the first compaction, the second and third pouring of mixture with the same weight as the first were, respectively, conducted in the same manner as the first.
3. After the third compaction, the height and weight of the mixture in the vessel were measured, and the volume of the mixture could be calculated. To ensure the accuracy of test results, at least two parallel tests were conducted, and when the difference between the parallel test data was larger than 5%, it was repeated. The VCA value of each sample was calculated according to Equations (1)–(3).

(1) $$\rho =\frac{M_{2}-M_{1}}{V}$$

where ρ is the accumulated density of the coarse aggregate (g/cm^3^); M_2_ is the total mass of the vessel and sample (g); M_1_ is the mass of the vessel (g); and V is the volume of the sample in vessel (mL).

(2) $$\rho_{b}=\frac{m_{1}{+\text{…}+m}_{n}}{\frac{m_{1}}{\rho_{1}}\text{…}+\frac{m_{n}}{\rho_{4}}}\;$$

where ρ_b_ is the synthetic bulk density of hybrid coarse aggregates (g/cm^3^); m_1_, …, m_n_ are the mass of various coarse aggregates (g), *n* = 4 in this study; and ρ_1_, …, ρ_n_ are the gross bulk density of various coarse aggregates (g/cm^3^).

(3) $$\mathrm{VCA}=\left(1\;-\frac{\rho }{\rho_{b}}\right)\text{×}100$$

## 3. Experimental Results and Discussion

### 3.1. Establishment of the Prediction Equation

The calculation results of the VCA of 16 kinds of hybrid coarse aggregate mixture samples are shown in Table 3.

Stepwise regression analysis of the experimental data in Table 3 was performed using MATLAB software. Since the proportion of coarse aggregate of each particle size in the mixture sums to 100 (i.e., x_1_ + x_2_ + x_3_ + x_4_ = 100), and one of the four variables must not be independent, so x_1_, x_2_, x_3_, and x_4_ should be, respectively, removed, then a stepwise regression equation was obtained between the VCA and three independent variables with the stepwise regression command in MATLAB (α = 0.05). The relative errors between the predicted and measured values of regression equations in each of the four cases were compared, and significance tests for the F-value were conducted for each equation. The regression equation with a smaller relative error, larger F-value, and the most significant value was selected as the statistical model between the VCA formed by hybrid lithological coarse aggregates and the proportion of each coarse aggregate.

**Figure 3:**
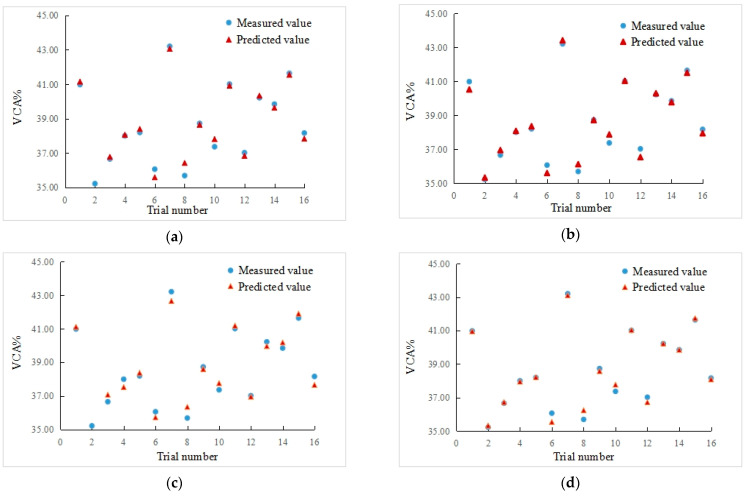
Comparisons between the measured and predicted values for each model: (**a**) regression Equation (4), (**b**) regression Equation (5), (**c**) regression Equation (6), and (**d**) regression Equation (7).

Regression equations obtained without considering x_1_, x_2_, x_3_, and x_4_ are shown in Equations (4)–(7), and the predicted values, average relative errors, R^2^, and F-values of each regression equation are shown in Table 4. The comparison between the measured value and predicted value of each regression equation are shown in Figure 3.

(4) $${\mathrm{VCA}}_{1}=45.2385\;-\;0{.52307x}_{4}-\;0{.0959748x}_{3}+0{.00320131x}_{3}x_{4}+0{.000709621x}_{3}^{2}\;\;+0{.00592327x}_{4}^{2}+0{.00179438x}_{2}x_{4}$$

(5) $${\mathrm{VCA}}_{2}=46.928\;-\;0.13297x_{3}-\;0.0349989x_{1\;}-\;0.43401x_{4}+0.00316946x_{3}x_{4}\;\;+0.00439545x_{4}^{2}+0.000862123x_{3}^{2}$$

(6) $${\mathrm{VCA}}_{3}=41.8458\;-\;0.259045x_{4}+0.000432029x_{1}^{2}-\;0.00198849x_{1}x_{4}\;\;+0.00043411x_{2}^{2}+0.00370339x_{4}^{2}$$

(7) $${\mathrm{VCA}}_{4}=49.1126\;-\;0.317215x_{3}-\;0.551397x_{1}-\;0.451033x_{2}+0.00906226x_{1}x_{2}\;\;+0.00607512x_{2}x_{3}+0.0064547x_{1}x_{3}+0.00512781x_{1}^{2}+0.00422807x_{2}^{2}+0.00249407x_{3}^{2}$$

It can be seen from the analysis results in Table 4 that the F-values of all established equations are substantially larger than F_critical_-values, indicating that all equations are significant at the significance level α = 0.05. Equation (7) has a minimum average relative error and a maximum R^2^; however, it has the most equation terms and a minimum F-value. Therefore, it is not recommended for further use. Equation (5) has a maximum F-value, a higher R^2^ value, a smaller average relative error, and fewer equation terms. Comprehensively considered, Equation (5) was selected as the prediction model for the VCA of hybrid lithological coarse aggregates.

According to the above equations, there is a multivariate nonlinear relationship between VCA and the proportion of each coarse aggregate. In addition, an interaction relationship exists, with an interference effect between aggregates of adjacent particle sizes (e.g., x_3_, x_4_) and a filling effect between aggregates with large differences in particle size (e.g., x_1_, x_4_).

### 3.2. Validation and Application of the Prediction Equation

#### 3.2.1. Validation of the Prediction Equation

**Figure 4:**
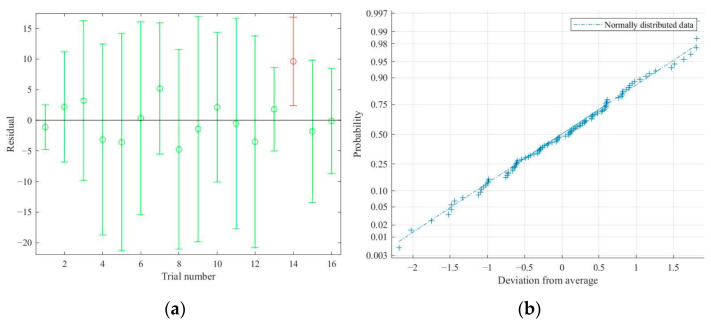
Residuals and normal probability plots of Equation (5): (**a**) residuals plots and (**b**) normal probability plots.

Significance testing and the comparison of average relative errors for Equation (5) were conducted in the above. To further validate the prediction equation, the residual plot and the normal probability plot were drawn by MATLAB software, shown in Figure 4.

From Figure 4, it can be seen that most residuals are distributed around the top and bottom of one straight line, and the normal probability plots form a straight line. So, the residuals of Equation (5) are random and obey normal distribution; thus, this prediction model of VCA formed by hybrid lithology aggregates is valid.

#### 3.2.2. Relationship between VCA and the Gradation Curve of Coarse Aggregates

The VCA prediction model of hybrid lithology aggregates revealed the changing law of VCA formed by mixing the coarse aggregates of different proportions (i.e., gradation curve), indicating that the VCA value is closely related to the shape of the gradation curve. The value of VCA has an effect on other volumetric indicators such as VMA, so the prediction model of VCA can be used to guide aggregate grading design for SMA. For example, in the design of initial grading for SMA, it is generally necessary to draw three grading curves within the standard grading range, then volume parameters for each grade of SMA mixture are determined by sample preparation, volume parameters testing, calculation, and so on. Using this model, it is possible to estimate the relative value of VCA of grading curves and the VMA value of the SMA mixture, allowing for an appropriate initial grading curve in accordance with design requirements, which can reduce experimental failures and workload.

**Figure 5:**
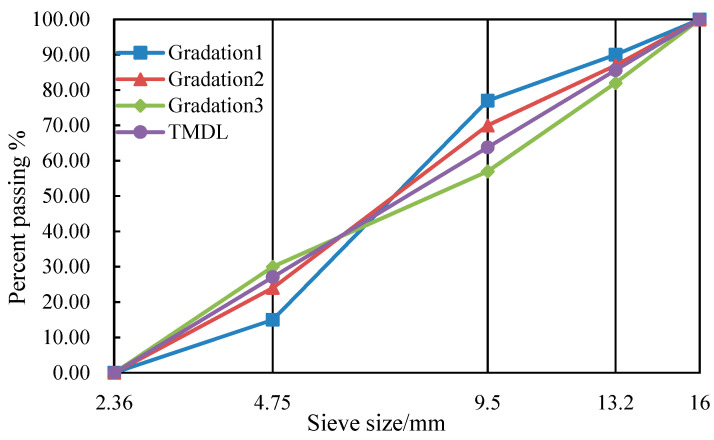
Gradation curves.

Further, the literature [19] gave the relationship between VCA and the shape of the grading curve of basalt coarse aggregate. However, whether the hybrid lithology aggregate follows the same law needs to be further analyzed and verified. For this purpose, four curves with typical characteristics were designed using coarse aggregate grading data from the literature [19], including two forward S-shaped grading curves, a reverse S-shaped curve, and a theoretical maximum density line (TMDL), as shown in Figure 5. Accordingly, the experimental method and the equation in this study were employed to measure and predict the VCA values of hybrid lithological coarse aggregate gradations and the results are presented in Table 5.

According to Table 5, the VCA values in descending order are Gradation 1 > Gradation 2 > TMDL > Gradation 3, in terms of the predicted and measured values. This indicates that the forward S-shaped grading curve has a higher VCA value, and the further the distance from the TMDL, the larger the VCA value; the reverse S-shaped gradation curve has a lower VCA value. Thus, the changing law between the shape of the hybrid lithological coarse aggregate grading curve and the VCA follows that reported in the literature [19]. It also verifies that the equation established in this paper is of general significance and can be used to predict the relative value of VCA of different mineral aggregate gradations, which is feasible and reasonable to guide the grading design of SMA with hybrid lithological aggregates.

## 4. Conclusions

In this paper, the uniform design method and the laboratory vibrating compaction experiment of coarse aggregate were applied to test the VCA formed by mixing two kinds of coarse basalt aggregates (13.2–16 mm, 9.5–13.2 mm) and two kinds of limestone aggregates (4.75–9.5 mm, 2.36–4.75 mm), which are used in SMA. A VCA prediction model was developed, and the variation of the VCA with the mineral aggregate grading curve was analyzed. The following conclusions can be drawn:

1. It is feasible and reliable to establish the VCA prediction equation for the hybrid lithological coarse aggregate by the uniform design and multiple regression analysis methods;
2. The VCA of the hybrid lithology coarse aggregate shows a multivariate nonlinear relationship with the proportion of aggregate of each particle size. There is an interference effect between adjacent particle size of the coarse aggregate and a filling effect between two grades of coarse aggregates with a larger particle size difference;
3. The rule between VCA and the aggregate gradation curves for different lithological coarse aggregates has universal significance. It has been further verified that the forward S-shaped gradation curve has a larger VCA value, while the reverse S-shaped curve has a smaller VMA value compared with the VCA of the TMDL.

## Figures and Tables

**Table 1:** Basic properties of coarse aggregates.

Properties	Test Values of Basalt		Test Values of Limestone		Specifications
	13.2~16 mm	9.5~13.2 mm	4.75~9.5 mm	2.36~4.75 mm	
Apparent specific gravity	2.890	2.892	2.798	2.801	>2.60
Bulk specific gravity	2.797	2.798	2.597	2.691	>2.50
Water absorption/%	1.15	1.13	1.20	1.21	≤2.0
Crushed stone value/%	12.0	19.9	-	-	≤26
L.A. abrasion/%	8.7	18.6	-	-	≤28
Percent of flat and elongated particles/%	9.8	10.2	13.6	-	≤15
Polished stone value (PSV)	51	45	-	-	≥40

**Table 2:** Uniform design scheme of different lithological coarse aggregate mixtures.

No.	x_1_/%	x_2_/%	x_3_/%	x_4_/%
1	7.8	8.9	14.8	68.5
2	33.9	1.1	10.4	54.6
3	20.8	30.4	2.6	46.1
4	21.6	2.2	36.4	39.7
5	15.4	29.4	20.7	34.5
6	52.2	9.7	8.1	29.9
7	3.7	9.4	61.0	25.9
8	35.6	10.0	32.1	23.3
9	14.4	51.3	15.4	19.0
10	59.5	23.3	1.3	16.0
11	6.4	34.2	46.3	13.1
12	42.8	22.4	24.2	10.4
13	7.9	76.7	7.5	7.9
14	17.2	11.8	65.6	5.5
15	1.9	59.8	35.1	3.2
16	44.6	39.3	15.1	1.1

**Table 3:** VCA values of hybrid coarse aggregates.

No.	Test Value/%		Average Value/%	No.	Test Value/%		Average Value/%
	(1)	(2)			(1)	(2)	
1	40.68	41.28	40.98	9	38.85	38.60	38.72
2	35.26	35.18	35.22	10	37.52	37.21	37.37
3	36.40	36.91	36.66	11	41.00	41.02	41.01
4	38.37	37.63	38.00	12	37.41	36.63	37.02
5	38.53	37.87	38.20	13	40.32	40.11	40.22
6	35.88	36.25	36.06	14	39.84	39.85	39.84
7	43.62	42.78	43.20	15	41.26	42.01	41.64
8	35.82	35.56	35.69	16	37.79	38.53	38.16

**Table 4:** Analytical parameters of regression equations.

Regression Equation No.	Average Relative Error/%	F	F_critical_	R^2^
(4)	0.62	87.292	3.374	0.983107
(5)	0.61	88.185	3.374	0.983275
(6)	0.83	76.338	3.326	0.97447
(7)	0.59	59.735	4.099	0.988963

**Table 5:** Predicted and measured VCA values of gradation curves.

No.	x_1_/%	x_2_/%	x_3_/%	x_4_/%	Measured Value/%	Predicted Value/%
Gradation 1	10.00	13.00	62.00	15.00	39.31	39.07
Gradation 2	13.00	17.00	46.00	24.00	37.57	37.80
Gradation 3	18.00	25.00	27.00	30.00	37.06	36.84
TMDL	14.36	21.85	36.71	27.08	37.11	37.33

## Data Availability

Data will be made available on request.
